# Beyond the Lungs: Cardiovascular Risk in COPD Patients with a History of Tuberculosis—A Narrative Review

**DOI:** 10.3390/jcm15020661

**Published:** 2026-01-14

**Authors:** Ramona Cioboata, Mihai Olteanu, Denisa Maria Mitroi, Simona-Maria Roșu, Maria-Loredana Tieranu, Silviu Gabriel Vlasceanu, Simona Daniela Neamtu, Eugen Nicolae Tieranu, Rodica Padureanu, Mara Amalia Balteanu

**Affiliations:** 1 Department of Pneumology, University of Medicine and Pharmacy, 200349 Craiova, Romania; ramona_cioboata@yahoo.com (R.C.); mihai.olteanu@umfcv.ro (M.O.); rodica.padureanu@umfcv.ro (R.P.); 2 Department of Pneumology, Victor Babes University Hospital, 200349 Craiova, Romania; 3 Doctoral School, University of Medicine and Pharmacy, 200349 Craiova, Romania; denisa_maria2@yahoo.com (D.M.M.); simona.vulpe1995@gmail.com (S.-M.R.); 4 Department of Obstetrics and Gynecology, Emergency County Hospital Craiova, 200642 Craiova, Romania; 5 Department of Microbiology, “Carol Davila” University of Medicine and Pharmacy, 050474 Bucharest, Romania; 6 Department of Immunology, Hematology, University of Medicine and Pharmacy of Craiova, 200349 Craiova, Romania; simona.neamtu@umfcv.ro; 7 Department of Internal Medicine-Cardiology, University of Medicine and Pharmacy, 200349 Craiova, Romania; eugen.tieranu@umfcv.ro; 8 Department of Pulmonology, Faculty of Medicine, Titu Maiorescu University, 031593 Bucharest, Romania; mara.balteanu@prof.utm.ro

**Keywords:** TB history, COPD, cardiovascular risk, biomarkers, QRISK4, obstructive sleep apnea, LMICs

## Abstract

Chronic obstructive pulmonary disease (COPD) and tuberculosis (TB) increasingly co-occur in low- and middle-income countries and aging populations. Prior pulmonary TB is a robust, smoking-independent determinant of COPD and is linked to persistent systemic inflammation, endothelial dysfunction, dyslipidemia, and hypercoagulability axes that also amplify cardiovascular disease (CVD) risk. We conducted a targeted narrative non-systematic review (2005–2025) of PubMed/MEDLINE, Embase, Scopus, and Web of Science, selecting studies for clinical relevance across epidemiology, clinical phenotypes, pathobiology, biomarkers, risk scores, sleep-disordered breathing, and management. No quantitative synthesis or formal risk-of-bias assessment was performed. Accordingly, findings should be interpreted as a qualitative synthesis rather than pooled estimates. Prior TB is associated with a distinctive COPD phenotype characterized by mixed obstructive–restrictive defects, reduced diffusing capacity (DLCO), radiographic sequelae, and higher exacerbation/hospitalization burden. Mechanistic insights: Convergent mechanisms chronic immune activation, endothelial injury, prothrombotic remodeling, molecular mimicry, and epigenetic reprogramming provide biologic plausibility for excess CVD, venous thromboembolism, and pulmonary hypertension. Multimarker panels spanning inflammation, endothelial injury, myocardial strain/fibrosis, and coagulation offer incremental prognostic value beyond clinical variables. While QRISK4 now includes COPD, it does not explicitly model prior TB or COPD-TB outcomes, but data specific to post-TB cohorts remain limited. Clinical implications: In resource-constrained settings, pragmatic screening, prioritized PAP access, guideline-concordant pharmacotherapy, and task-shifting are feasible adaptations. A history of TB is a clinically meaningful modifier of cardiopulmonary risk in COPD. An integrated, multimodal assessment history, targeted biomarkers, spirometry/lung volumes, DLCO, 6 min walk test, and focused imaging should guide individualized care while TB-aware prediction models and implementation studies are developed and validated in high-burden settings.

## 1. Introduction

Chronic obstructive pulmonary disease (COPD) and tuberculosis (TB) are both major global health challenges, especially in low- and middle-income countries (LMICs) and among aging populations [1]. Between 2007 and 2017, global mortality attributable to COPD increased by 17.5%, reaching approximately 3.2 million deaths in 2017 [2]. In 2019, there were over 212 million cases and 3.3 million deaths globally, with more than 75% of cases in LMICs [3,4].

The burden is projected to rise, especially in LMICs and among older adults, due to population growth, aging, and ongoing exposure to risk factors like smoking and air pollution. These drivers may also elevate COPD incidence among non-smokers, with implications for both prevention and treatment strategies [5]. Globally, COPD prevalence is estimated at 10.3% (95% CI: 8.2–12.8) [1,6]. Projections from the Global Burden of Disease indicate that among adults ≥ 25 years, COPD cases could rise by ~23% between 2020 and 2050, approaching 600 million by 2050 [7]. This growth is expected to disproportionately affect women and populations in low- and middle-income countries.

Tuberculosis (TB) remains a leading infectious killer, causing ~10 million new cases and 1.2 million deaths annually, with the highest burdens in South-East Asia and Africa, predominantly across LMICs. In 2023, an estimated 8.2 million individuals were newly diagnosed with tuberculosis up from 7.5 million in 2022 and 7.1 million in 2019, and well above the 5.8 million and 6.4 million reported in 2020 and 2021, respectively. This resurgence likely reflects the clearance of a backlog of undetected cases accumulated during the COVID-19 pandemic, when disruptions to health services delayed TB diagnosis and treatment [8,9].

Its intersection with COPD is increasingly consequential: TB survivors face a 1.7- to 4-fold increased risk of developing COPD, independently of smoking and other common risk factors [10,11,12], and display significantly reduced lung function parameters, such as forced expiratory volume (FEV_1_) and forced vital capacity (FVC), compared to those without prior TB [13].

Nearly half of TB’s lifetime disease burden is attributable to post-TB sequelae most notably chronic respiratory impairment and COPD which may persist for decades after microbiological cure [11]. Because LMICs shoulder more than 75% of global COPD cases and have limited capacity for diagnosis and long-term management, improvements in TB survival coupled with population aging portend rapid growth in post-TB COPD, further straining health systems [7]. Shared risk factors including smoking, poverty, and ambient and household air pollution compound disability, degrade quality of life, and impose substantial macroeconomic losses; LMICs account for an estimated 56% of the global economic burden from COPD [14,15].

Post-tuberculosis lung disease (PTLD) is increasingly recognized as a heterogeneous, long-term consequence of TB that encompasses overlapping airway, parenchymal, and vascular abnormalities, affecting millions of survivors worldwide. Airway sequelae commonly include bronchiectasis, tracheobronchial stenosis, chronic airflow obstruction, and broncholithiasis, leading to persistent cough, wheeze, recurrent infections, and vulnerability to superinfections such as chronic pulmonary aspergillosis [16]. Parenchymal injury characterized by fibrosis, cavitation, and tuberculomas produces restrictive or mixed ventilatory defects, reduced lung volumes, impaired gas exchange, and imaging evidence of architectural distortion and volume loss [17]. Vascular complications, though less frequent, span Rasmussen aneurysms, pulmonary or bronchial arteritis, thrombosis, and pulmonary hypertension, with presentations ranging from life-threatening hemoptysis to rare compressive neuropathies such as Ortner’s syndrome [18]. Recognizing PTLD as a spectrum rather than a single entity is essential for accurate diagnosis, tailored management, and improved long-term outcomes in TB survivors. In patients with COPD and prior TB, the clinical phenotype is typically more severe coexisting obstruction and restriction, lower DLCO, and greater exertional desaturation on the 6 min walk test features that track with higher exacerbation rates and poorer functional status. TB-modified COPD also signals heightened cardiovascular vulnerability: observational cohorts report increased hypertension, ischemic heart disease, stroke, venous thromboembolism, and right-heart strain, plausibly mediated by persistent systemic inflammation, endothelial injury, and a prothrombotic state. Recognizing PTLD as a spectrum and the COPD–post-TB phenotype as a distinct high-risk state supports an integrated cardiopulmonary assessment and earlier cardiovascular risk mitigation in TB survivors [19,20,21].

For clarity, PTLD is an umbrella term for persistent symptoms and/or structural/functional lung abnormalities after TB treatment. TB-associated COPD denotes COPD-defined chronic airflow limitation in individuals with prior TB, whereas classic smoking-related COPD is predominantly driven by long-term tobacco exposure and often includes emphysema/chronic bronchitis phenotypes. These entities can overlap, but distinguishing them helps interpret mechanisms and cardiopulmonary risk.

This narrative review aims to synthesize and critically appraise the evidence on how prior tuberculosis modifies cardiopulmonary risk in individuals with COPD and, by systematically mapping current knowledge and gaps, to provide a coherent framework that guides comprehensive cardiopulmonary care for COPD patients with a history of TB while prioritizing directions for future research and guideline development. In this review, we use cardiopulmonary risk to refer to respiratory and pulmonary vascular outcomes, including diffusing capacity for carbon monoxide (DLCO), 6 min walk test (6MWT) nadir SpO_2_, pulmonary hypertension (PH), venous thromboembolism (VTE), and COPD exacerbations. We use cardiovascular (CVD) risk to refer to systemic cardiac and arterial events, including myocardial infarction (MI), stroke, heart failure (HF), and arrhythmias. When outcomes span both domains, we explicitly specify the primary endpoint under consideration.

## 2. Search Strategy

To enhance transparency and reproducibility, we conducted a targeted search of PubMed/MEDLINE, Embase, Scopus, and Web of Science for studies published from 2005 through 2025. The search combined controlled vocabulary and free-text terms related to “COPD”, “TB history”, “post-tuberculosis lung disease”, “cardiovascular events”, “QRISK4”, “QRISK3”, “cardiovascular risk”, “exacerbation”, “heart failure”, “myocardial infarction”, “heart failure”, “thromboembolism”, “novel biomarkers”.

**Figure 1:**
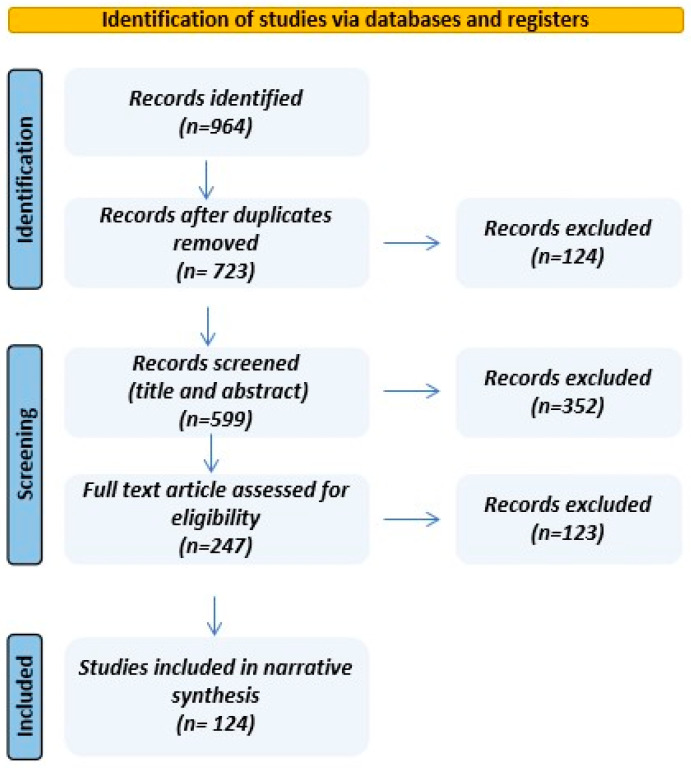
Descriptive flow diagram for study selection.

Only English-language research, systematic reviews, meta-analyses, and narrative reviews were eligible; studies lacking sufficient methodological or outcome detail or falling outside the date range were excluded. After removing duplicates, titles and abstracts were screened, followed by full-text assessment to determine inclusion. A descriptive flow diagram summarizes study selection (Figure 1). Given our narrative aim, evidence was synthesized based on relevance and clinical applicability rather than systematic data extraction; accordingly, no formal risk-of-bias appraisal or quantitative meta-analysis was undertaken.

When multiple reports addressed the same question, we prioritized studies with larger sample sizes, clearer case definitions for PTLD/TB history and COPD diagnostic criteria, longer follow-up, and outcomes with direct clinical significance. Clinical relevance was operationalized a priori as evidence directly informing cardiopulmonary or cardiovascular outcomes in adults with COPD and/or a history of TB, including hard clinical endpoints such as mortality, exacerbations, hospitalization, myocardial infarction, stroke, heart failure, pulmonary hypertension, and venous thromboembolism, validated surrogate or functional measures including DLCO, 6MWT performance and nadir SpO_2_, and echocardiographic estimates of pulmonary pressures, and management implications applicable to routine care. We preferentially considered population-based cohorts and randomized trials when available, and used high-quality systematic reviews/meta-analyses primarily for background context, while leveraging mechanistic studies to support biological plausibility.

## 3. Epidemiology and Clinical Phenotypes

Prior TB is an important and regionally variable determinant of COPD risk. Across multicounty cohorts in LMICs, the prevalence of prior TB among individuals with COPD averages ~2.7%, with site-specific estimates spanning 1.7–15.5% [10,11]. In these settings, TB history is also associated with a markedly higher probability of COPD among the general population (≈25.7% in those with prior TB vs. 8.3% without TB) [10,11]. Similar patterns are observed in East and South Asia. In China, population-based data indicate a prior TB prevalence near 2.7%, yet COPD occurs disproportionately among those with past TB (21.8% vs. 11.9% in those without TB) [12]. In India, hospital-based cohorts from high-burden regions report that roughly one-third of COPD patients have a documented TB history, with estimates ranging from 11% to 58% across studies [22,23]. Pooled evidence from meta-analyses further supports this gradient, placing the global prevalence of COPD among individuals with prior TB at ~21% (95% CI, 16–25%), despite considerable heterogeneity by study design and geography [24]. These regional contrasts are summarized in Table 1.

Importantly, emerging incidence data suggest this association may be time dependent. In the work of Joo et al., the incidence of tuberculosis-associated COPD in the longest time-since-TB quartile (>132 months, ~11 years) was significantly lower than in the earliest quartile (18–71 months), with an incidence rate ratio of 0.49 (*p* = 0.027), implying that excess risk is greatest in the first years after TB and may attenuate over time [29].

In a population-based matched cohort study from Korea, Kim et al. reported that tuberculosis survivors had a significantly increased risk of incident COPD and COPD-related hospitalizations compared with matched controls [30].

Beyond prevalence, prior TB confers a substantial, smoking-independent increase in COPD risk on the order of 1.6- to 3.8-fold in adjusted analyses, an effect that appears strongest in high TB-burden settings and among never-smokers [10,11,12]. Clinically, COPD patients with a history of TB experience more severe respiratory symptoms, greater impairment in lung function, and higher rates of exacerbations and hospitalizations than their counterparts without TB. Collectively, these data position prior TB as a major, context-dependent contributor to COPD burden worldwide and underscore the need to integrate TB prevention and post-TB care into COPD risk stratification and management pathways, particularly in high-incidence regions [13].

Tuberculosis-associated COPD (TB-COPD) is increasingly recognized as a distinct phenotype that differs meaningfully from smoking-related COPD in onset, structure, inflammation, and clinical course. Patients typically present at a younger age with more severe airflow limitation, greater symptom burden (notably dyspnea, cough, and hemoptysis), and a higher frequency of exacerbations and hospitalizations [31,32]. Imaging and pathological hallmarks include prominent bronchiectasis localized to prior TB lesions, more extensive middle- and lower-lobe emphysema with panlobular/centrilobular patterns, and frequent fibrosis, cavitation, and airway remodeling consistent with chronic post-infectious damage [33,34]. Biologically, TB-COPD shows heightened systemic and airway inflammation such as elevated IL-6 and CRP, persistent immune activation, and distinct metabolic signatures such as altered fatty-acid and tryptophan pathways paralleling its increased susceptibility to secondary infections. These differences have direct therapeutic implications: while long-acting bronchodilators and muscarinic antagonists remain foundational, clinicians should individualize care, weigh inhaled corticosteroid use against TB reactivation risk, and prioritize strategies tailored to the post-TB airway and parenchyma, particularly in high-burden settings [12,35,36]. Recognizing TB-COPD as a discrete entity is therefore critical for diagnosis, risk stratification, and management.

**Figure 2:**
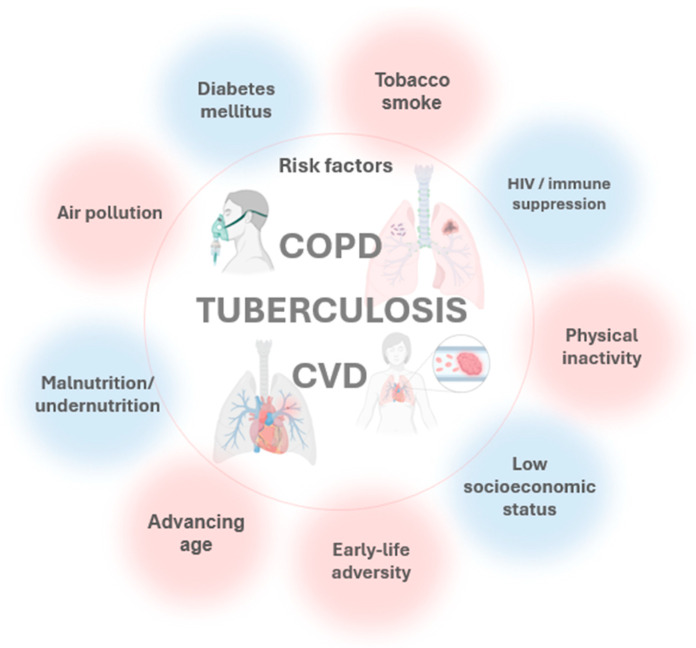
Risk factors for COPD, TB, and CVD.

Smoking intensity, biomass exposure, HIV coinfection, diabetes, and malnutrition are key confounders and effect modifiers in the TB-COPD relationship, independently shaping risk, phenotype, and outcomes. Beyond these modifiers, COPD, TB, and CVD arise from a common exposure profile tobacco and air pollution within contexts of socioeconomic disadvantage, metabolic disease, immunosuppression, physical inactivity, and adverse early-life factors and converge on a limited set of pathobiological axes: persistent inflammation; oxidative/mitochondrial stress; endothelial dysfunction with impaired nitric-oxide signaling; immune–metabolic dysregulation; prothrombotic activation; autonomic imbalance; protease–antiprotease and matrix remodeling; microbiome perturbation; and accelerated biological aging [37,38,39]. This shared network plausibly links post-infectious and chronic lung injury to atherothrombosis, pulmonary hypertension, and other cardiovascular events, explaining overlapping symptoms and underscoring the need for integrated, confounder-aware risk assessment (Figure 2).

Multiple studies highlight that smoking is the most prominent shared risk factor for TB, COPD, and CVD. Smoking increases susceptibility to TB, is the leading cause of COPD, and is a well-established risk factor for CVD [40]. Heavy smoking amplifies the risk of TB, worsens airflow obstruction, and increases the likelihood of cavitary TB lesions and poor outcomes in both diseases [41,42,43]. However, prior TB remains an independent risk factor for COPD even after adjusting for smoking, and smoking does not significantly modify the TB-COPD association [13,44].

Biomass smoke exposure is a significant, independent risk factor for both COPD and TB, especially in women and non-smokers in LMICs. Biomass exposure increases COPD risk (OR 2.44–3.2) and is associated with higher ICU admissions and mortality in COPD patients. The effects of biomass and tobacco smoke are additive, and both exposures are linked to distinct inflammatory profiles and disease phenotypes [45,46,47].

HIV infection heightens susceptibility to both TB and COPD, with synergistic effects that translate into greater morbidity and mortality than either condition alone [43]. Diabetes further compounds this risk landscape: it is linked to increased incidence and severity of TB and is associated with poorer COPD outcomes; importantly, tobacco use markedly amplifies TB risk among individuals with diabetes, with odds ratios reported up to 7.6 [41]. Malnutrition prevalent across TB and COPD populations acts as a shared, upstream determinant that exacerbates immune dysfunction, may contribute to disease progression, and worsens clinical outcomes in both disorders [26,48].

Prior tuberculosis is strongly linked to increased long-term cardiopulmonary risk, with chronic inflammation, immune activation, and epigenetic changes as key pathobiological mechanisms.

### 3.1. Chronic Inflammation and Immune Activation

TB history amplifies CVD risk through convergent immune–vascular pathways. Active infection sustains monocyte–macrophage and lymphocyte activation with heightened TNF-α, IFN-γ, IL-1, and IL-6 signaling, a cytokine milieu that mirrors and may contribute to atherogenesis by fostering plaque initiation and progression [49,50]. The resulting endothelial activation increases expression of adhesion molecules such as VCAM-1 and ICAM-1, enhancing leukocyte adhesion and transmigration, perpetuating vascular inflammation, and compromising nitric oxide–mediated vasodilation early steps in atherogenesis and vascular injury. Notably, even latent TB infection is associated with persistent low-grade inflammation (e.g., elevated CRP) and immune activation that correlate with higher risks of hypertension, insulin resistance/diabetes, and subclinical atherosclerosis [51,52]. Superimposed autoimmune mechanisms, including molecular mimicry whereby antibodies to mycobacterial antigens cross-react with vascular self-epitopes, may further amplify endothelial injury and plaque development, providing a biologically coherent link between TB and downstream cardiovascular events [53]. Chronic inflammation and immune activation from TB infection, active or latent, are central to the development of atherosclerosis, endothelial dysfunction, and vascular injury, explaining the increased cardiovascular risk observed in TB survivors. This link is supported by both mechanistic and epidemiological research.

### 3.2. Endothelial Dysfunction and Coagulopathy

TB is associated with a vascular milieu characterized by endothelial dysfunction and hypercoagulability that appears during active disease and can persist after microbiologic cure. Systemic inflammation perturbs endothelial homeostasis altering VEGF, nitric oxide, endothelin-1, and von Willebrand factor (vWF), thereby increasing permeability, dampening endogenous anticoagulant pathways, and priming platelet adhesion [54,55,56]. Notably, biomarkers of endothelial injury and impaired nitric oxide signaling remain elevated following treatment especially in individuals with multiple prior TB episodes implicating durable vascular remodeling and heightened susceptibility to pulmonary hypertension [38]. In parallel, TB-driven cytokine signaling upregulates tissue factor, enhances platelet activation, and suppresses anticoagulant and fibrinolytic mechanisms, culminating in a hypercoagulable state with increased risk of venous thromboembolism, including deep vein thrombosis and pulmonary embolism [37]. Emerging genetic data further suggest that host variants governing endothelial biology and platelet homeostasis modulate TB severity and may compound thrombotic risk. Collectively, these immune–vascular perturbations provide a mechanistic basis for the excess burden of thrombotic and long-term cardiovascular complications observed in TB survivors. TB infection, both active and post-treatment, is associated with persistent endothelial dysfunction and a hypercoagulable state, significantly increasing the risk of thrombotic events and vascular complications. Monitoring and managing these risks is crucial for improving long-term outcomes in TB patients.

### 3.3. Autoimmunity and Molecular Mimicry

Molecular mimicry between mycobacterial and human heat-shock proteins biologically consistent with an autoimmune pathway linking TB exposure to atherogenesis. Antibodies elicited against mycobacterial 65 kDa heat-shock protein (HSP65) can cross-recognize homologous epitopes on human HSP60 displayed by stressed arterial endothelium, breaching tolerance and directing humoral attack on vascular self-antigens [57,58,59]. These cross-reactive antibodies exert endothelial cytotoxicity, particularly under cellular stress, thereby amplifying vascular injury that seeds and may contribute to plaque formation [57]. In parallel, T cells primed by HSP65 may recognize shared self-epitopes, sustaining a pro-inflammatory vascular milieu through antigen-specific cellular immunity. A history of TB can perpetuate this axis by maintaining cross-reactive humoral and T-cell memory to HSP65/HSP60 via epitope spreading, residual antigen depots, and trained innate immunity, thereby sustaining endothelial injury and potentially accelerating atherogenesis well beyond microbiologic cure. Convergent human, mechanistic, and animal data are consistent with this mechanism, elevated anti-HSP65 antibodies are associated with carotid atherosclerosis and vasculitis independent of traditional risk factors, epitope-mapping identifies HSP65/HSP60-shared determinants within early human lesions, patient-derived anti-HSP65/60 antibodies damage endothelial cells in vitro, and in vivo, HSP65 immunization may contribute to arteriosclerotic lesions, whereas oral tolerance to HSP65 mitigates atherosclerosis [59,60]. There is strong evidence that immune responses to mycobacterial HSP65 can trigger autoimmunity via molecular mimicry, potentially leading to vascular inflammation and atherosclerosis. This process involves both antibody- and T cell-mediated mechanisms and is supported by human, animal, and molecular studies.

### 3.4. Direct Cardiac Involvement

Direct cardiac involvement in tuberculosis spans a spectrum in which pericarditis is most common particularly in endemic regions and among immunocompromised hosts manifesting as acute pericarditis, pericardial effusion, myopericarditis, or constrictive pericarditis; in these settings, TB remains the leading cause of constrictive physiology, and complications such as cardiac tamponade, heart failure, and death can ensue without timely therapy [39,61]. Myocarditis is far less frequent (prevalence < 2%) yet clinically consequential, presenting with acute or chronic heart failure, ventricular arrhythmias, and conduction disease, and is often underrecognized in life given its potential for hematogenous, lymphatic, or contiguous myocardial seeding findings frequently confirmed only at autopsy. Aortitis and coronary artery involvement are rarer still, but TB can precipitate aortitis with mycotic aneurysm formation or coronary arteritis, which can culminate in aneurysm rupture, myocardial infarction, or sudden death [39,61,62]. Other unusual entities such as endocarditis, intracardiac tuberculomas, and valvular disease have been reported but remain exceptional. Clinically, both pericardial and myocardial forms are important associations of heart failure and sudden cardiac death; the latter in particular has been linked to TB myocarditis in younger adults via malignant arrhythmias or fulminant pump failure. Moreover, COPD patients with a prior history of TB constitute a persistently high-risk subgroup, as residual pericardial/myocardial scarring, post-TB pulmonary vascular disease, and sustained endothelial–coagulopathic activation can maintain an elevated baseline risk of decompensation, arrhythmic events, and heart failure even after microbiological cure [63]. Early recognition is critical: echocardiography and advanced imaging support diagnosis and phenotyping, while prompt initiation of anti-tuberculous therapy and, when indicated, surgical interventions such as pericardiectomy are pivotal to avert adverse outcomes [64,65,66].

### 3.5. Epigenetic Changes

Emerging epigenomic evidence suggests that TB may leave a durable molecular imprint on cardiometabolic pathways. In a 2025 pilot study among people with HIV, both prior active and latent TB were associated with thousands of differentially methylated CpG sites/regions in peripheral blood, with prior active TB showing significant enrichment for pathways implicated in vascular smooth muscle contraction and inherited cardiomyopathies (arrhythmogenic right ventricular, hypertrophic, dilated), implying that TB may drive lasting methylation shifts in cardiovascular gene networks [67]. These findings align with broader syntheses showing that DNA methylation and related epigenetic modifications regulate key axes of atherosclerosis, inflammation, hypertension, and vascular smooth muscle cell (VSMC) function, and that environmentally driven methylation changes including those triggered by infections track with elevated cardiovascular risk [68,69]. Mechanistic work further supports biological plausibility: methylation remodeling in VSMCs can impair contractile phenotype and promote vascular dysfunction, providing a credible conduit through which TB-induced epigenetic alterations might translate into downstream cardiovascular disease. Current research supports the theory that prior TB infection can cause persistent DNA methylation changes in cardiovascular pathways, providing a plausible epigenetic mechanism for increased long-term CVD risk in TB survivors.

### 3.6. Medication-Related Modifiers

The management of TB entails multidrug regimens whose direct toxicities, pharmacokinetic interactions, and adjunctive strategies can meaningfully modify cardiopulmonary risk. Notably, bedaquiline, delamanid, and several fluoroquinolones prolong the QT interval and may precipitate malignant arrhythmias risks that compound when multiple QT-prolonging agents are co-administered or in patients with elevated Tisdale scores [39]. COPD patients with a prior history of tuberculosis constitute a persistently high-risk subgroup of residual parenchymal and vascular sequelae, ongoing systemic inflammation, and autonomic dysregulation maintain an elevated baseline risk of decompensation and arrhythmic events, thereby amplifying the clinical impact of QT-prolonging regimens and drug–drug interactions even after microbiological cure [63]. Linezolid and clofazimine mainstays in drug-resistant TB carry predictable, usually reversible myelosuppression and peripheral neuropathy burdens that warrant proactive monitoring [70]. By contrast, pyrazinamide-related hyperuricemia is common but has not been linked to major adverse cardiovascular events and may even track with improved TB outcomes [71]. Among drug–drug interactions, rifampicin’s potent enzyme induction can blunt exposure to widely used cardiovascular and diabetes therapies including statins, sulfonylureas, calcium-channel blockers, ACE inhibitors, and warfarin, complicating care for patients with TB–diabetes or TB–CVD multimorbidity; in this context, metformin is generally preferred for glycemic control (insulin reserved for severe or unstable hyperglycemia) owing to minimal interaction with rifampicin [72,73,74]. Adjunctive, host-directed approaches are increasingly explored: statins may lower systemic inflammation and potentially potentiate antimycobacterial therapy but though confirmatory trials are needed [39,75], while aspirin in TB with diabetes improves inflammatory biomarkers and has been associated with better treatment outcomes [76]. Finally, corticosteroids and mTOR-pathway modulators are used selectively to temper hyperinflammation particularly in severe or extrapulmonary disease balancing potential cardiometabolic liabilities against benefits in organ protection and survival. Medication-related modifiers, including drug toxicities, interactions, and adjunctive therapies, play a significant role in shaping cardiopulmonary risk in TB patients. Careful selection, monitoring, and integration of therapies are essential to optimize outcomes and minimize cardiovascular complications.

## 4. Clinical Assessment and Risk Stratification

COPD patients with prior pulmonary TB represent a chronically high-risk phenotype: post-infectious parenchymal and vascular sequelae, coupled with persistent inflammation and prothrombotic activation, sustain elevated CVD risk after cure, arguing for risk models that explicitly account for COPD–TB overlap. Effective clinical assessment and risk stratification are important for identifying patients at increased risk of CVD and for optimizing treatment strategies. Recent research highlights both traditional and novel approaches to risk prediction in this population. In interpreting mechanistic pathways, it is important to distinguish direct evidence from COPD–TB cohorts from indirect evidence extrapolated from related contexts such as TB without COPD, COPD without TB, or broader inflammatory/cardiovascular literature. Where data are not derived from post-TB COPD populations, we present these mechanisms as hypothesis-generating and supportive of biological plausibility rather than definitive causal pathways in COPD–TB overlap.

### 4.1. History and Physical Examination

A meticulous history and examination are pivotal for cardiopulmonary risk stratification in TB survivors. Clinicians should document the exact treatment regimen and duration, including interruptions, switches for toxicity, or drug resistance-driven modifications, given their bearing on long-term outcomes [77]. The cumulative number of TB episodes should be recorded, as recurrent disease correlates with greater post-TB lung impairment and downstream cardiovascular complications [21,61]. Comorbid conditions, particularly diabetes, HIV/immunodeficiency, and any prior extrapulmonary or cardiac TB must be elicited because they amplify adverse cardiopulmonary trajectories. Post-treatment symptom clusters commonly include cough, sputum production, and exertional breathlessness; among these, dyspnea–desaturation mismatch (disproportionate exertional symptoms with marked oxygen desaturation) is a high-yield clinical signal of post-TB lung disease, pulmonary vascular involvement, or occult cardiac dysfunction [72,78]. The physical examination should target hemodynamic status such as tachycardia, hypotension, gas exchange (resting or exertional hypoxemia), and respiratory mechanics such as asymmetric chest excursion, crackles/rhonchi suggestive of bronchiectasis or fibrosis, while remaining vigilant for infrequent but life-threatening presentations such as pneumothorax or cardiac tamponade when acute dyspnea coexists with instability. Together, these history elements and bedside findings form the scaffold for subsequent testing and longitudinal risk management in TB-associated cardiopulmonary disease.

### 4.2. Pulmonary Function and 6MWT Insights

COPD patients with prior TB exhibit a distinct cardiopulmonary phenotype characterized by more severe and complex impairment of lung function and exercise capacity. Compared with COPD patients without TB, those with a TB history demonstrate more frequently combined obstructive and restrictive ventilatory defects on spirometry, alongside markedly reduced diffusing capacity (DLCO) reported in up to 78% of post-TB individuals reflecting structural destruction and persistent gas-exchange abnormalities [79,80]. Mixed patterns are common, and the extent of radiologic sequelae correlates with lower FEV_1_, FVC, and DLCO, underscoring the anatomical physiologic coupling of parenchymal damage and airway disease in this subgroup [20,81].

Functional assessment with the 6MWT provides complementary prognostic information in COPD with post-TB sequelae [19,82,83]. Nadir SpO_2_ during the 6MWT is often substantially reduced, mirroring impaired diffusion and ventilation–perfusion mismatch; among pulmonary function metrics, DLCO is the strongest independent predictor of exercise-induced desaturation, with DLCO < 45% predicted identifying patients at high risk for clinically meaningful SpO_2_ declines [80,84]. While the magnitude of desaturation relates variably to spirometric indices, 6MWT distance remains an independent predictor of mortality, reinforcing the value of integrated physiologic and functional profiling to stratify risk and guide longitudinal management in COPD patients with a history of TB [19].

### 4.3. Prognostic Biomarkers for Cardiovascular Risk Stratification in COPD

In COPD with cardiovascular comorbidities, circulating biomarkers are not screening tools but add clinically meaningful context for risk stratification, prognosis, and management. NT-proBNP/BNP reflects myocardial wall stress and cardiopulmonary strain from hypoxia and inflammation; higher levels track with greater severity, pulmonary hypertension or heart failure, acute exacerbations, and in-hospital mortality. High-sensitivity troponin (hs-TnT) indexes myocardial injury and independently predicts major adverse cardiovascular events and long-term mortality, with particularly strong prognostic yield when interpreted alongside NT-proBNP.

D-dimer signals activation of coagulation and fibrinolysis, aiding evaluation of venous thromboembolism and identifying patients with worse survival, especially when pretest probability for pulmonary embolism or cancer is elevated.

Inflammatory biomarkers high-sensitivity C-reactive protein (hs-CRP), fibrinogen, interleukin-6 (IL-6), and interleukin-8 (IL-8) capture complementary dimensions of vascular inflammation and injury and, in aggregate, improve cardiovascular risk stratification beyond traditional factors. Among these, IL-6 consistently emerges as a potent independent predictor of incident cardiovascular events, heart failure, and all-cause mortality, often outperforming hs-CRP and fibrinogen even after multivariable adjustment [85,86,87]. hs-CRP is widely validated for forecasting future CVD, heart failure, and mortality, with particularly robust performance when integrated into multimarker models or applied in statin-treated populations [88,89]. Fibrinogen confers additive prognostic value especially alongside hs-CRP with higher levels linking to acute coronary events and adverse outcomes. Although comparatively less studied, IL-8 independently predicts long-term cardiovascular events most notably in established coronary artery disease, highlighting a neutrophil-driven inflammatory axis that is not fully captured by other markers [90]. Collectively, multimarker approaches that combine IL-6, hs-CRP, fibrinogen, and IL-8 offer a more granular appraisal of inflammatory risk and may refine prevention and monitoring strategies in at-risk populations. Among COPD phenotypes, those with documented prior pulmonary TB show biomarker baseline shifts higher IL-6/hs-CRP/fibrinogen and D-dimer, with more frequent NT-proBNP or hs-troponin elevation at comparable clinical severity consistent with post-infectious remodeling of inflammatory, endothelial, and coagulation pathways. Accordingly, decision thresholds should be interpreted more conservatively, and TB history should be modeled as a covariate/interaction term in risk tools to improve calibration and reclassification [63].

### 4.4. Novel Biomarkers

**Figure 3:**
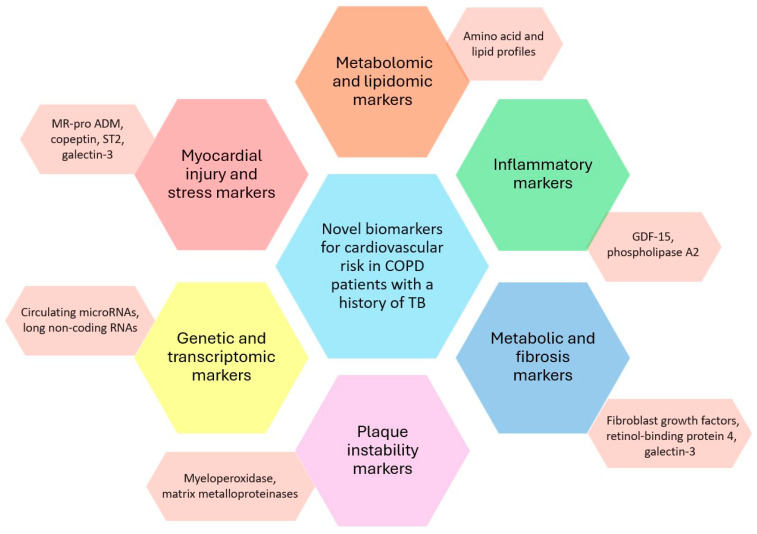
Predictive biomarkers for risk in COPD patients with a history of TB. GDF-15: growth differentiation factor-15; MR-proADM: mid-regional pro-adrenomedullin; ST2: the IL-33 receptor axis biomarker.

An expanding armamentarium of novel biomarkers is reshaping cardiovascular risk prediction by illuminating complementary axes of disease biology including inflammation and plaque instability, myocardial injury and hemodynamic stress, metabolic dysfunction and fibrosis, and gene-regulatory programs thereby extending diagnostic, prognostic, and therapeutic guidance beyond traditional factors (Figure 3).

Inflammatory/plaque markers such as growth differentiation factor-15 (GDF-15), myeloperoxidase, matrix metalloproteinases, lipoprotein-associated phospholipase A_2_, and secretory phospholipase A_2_ track vascular inflammation, cap disruption, and acute coronary syndromes [91,92].

Myocardial injury/stress markers; high-sensitivity cardiac troponins; heart-type fatty-acid–binding protein; cardiac myosin-binding protein-C; and natriuretic/related peptides (BNP, NT-proBNP, MR-proADM, copeptin, ST2, galectin-3) are validated for early infarct detection and granular heart-failure risk stratification [93].

Metabolic/fibrotic markers including adiponectin, fibroblast growth factors (FGF19/FGF21), retinol-binding protein-4, and galectin-3 capture maladaptive metabolism and extracellular-matrix remodeling with additive prognostic value [94]. Genetic/transcriptomic markers circulating microRNAs, long non-coding RNAs, and multi-omics signatures assembled with machine learning are emerging as sensitive, noninvasive tools for early detection, individualized risk staging, and therapeutic targeting [95]. Finally, multimarker and ML-enabled approaches leveraging aptamer-based proteomics and AI-driven analytics consistently outperform single-analyte strategies, enabling patient-specific phenotyping and more accurate event prediction.

Emerging data suggest that many of these biomarkers tend to be higher in COPD patients with prior TB, consistent with persistent immune activation, endothelial dysfunction, and prothrombotic tone observed after TB treatment. In particular, IL-6, hs-CRP, procalcitonin and fibrinogen frequently remain elevated beyond microbiologic cure, while IL-8 may capture residual neutrophil-driven airway and vascular inflammation; D-dimer and related coagulation markers can also be higher where post-TB vascular remodeling or venous thromboembolism risk is increased [91,92,96]. High monocyte levels independently predict poor prognosis and lower cure rates in TB patients, especially those with anemia, and may be relevant in COPD-TB overlap [97]. Blood-based gene expression profiles (e.g., Risk6, Risk16) can predict progression from latent to active TB and treatment failure, even in those with prior TB. These signatures show high sensitivity and specificity and are being validated for point-of-care use [98]. Altered serum levels of glutamate, methionine, asparagine, and specific lipid species are found in active TB and COPD, offering potential for noninvasive risk prediction and disease monitoring [99].

Accordingly, a history of TB in COPD patients should be treated as a biological modifier when interpreting biomarker values for cardiovascular risk: expected baselines may shift upward, multimarker panels may show greater discrimination than single analytes, and trajectory (change over time) and context (symptoms, imaging, pulmonary function, and comorbidities) become especially important. Importantly, multimarker approaches that integrate novel biomarkers with traditional risk factors outperform single biomarkers or clinical variables alone, offering a more granular assessment to guide triage, follow-up intensity, and preventive therapy.

### 4.5. Novel Integrated Prediction Scores

QRISK4 the latest iteration of the UK’s widely adopted 10-year CVD risk calculator extends prior QRISK models by integrating additional, novel determinants and improving sex-specific calibration. the formal inclusion of COPD acknowledges the cardiometabolic and vascular burden associated with chronic lung disease [100]. However, the accuracy of QRISK4 in individuals who have both COPD and a history of TB warrants caution: post-TB survivors often exhibit persistent inflammation, endothelial dysfunction, and altered cardiopulmonary physiology that may not be fully captured by conventional clinical variables. Studies show that QRISK3 (and by extension, QRISK4) significantly underestimates the actual 10-year CVD risk in patients with COPD. Observed CVD risk in COPD patients is up to 52% higher than predicted, and this gap is even greater in those under 65 years old [101].

Prior pulmonary TB independently increases the likelihood of developing COPD and is associated with persistent systemic inflammation, dyslipidemia, and endothelial dysfunction features that further amplify CVD risk beyond conventional factors. Individuals with COPD–TB overlap demonstrate more intense inflammatory and oxidative stress signatures and greater metabolic derangement, translating into higher rates of cardiovascular complications and premature mortality [102]. Consistent with this biology, patients with prior TB especially those with concomitant COPD exhibit elevated C-reactive protein and fibrinogen and display adverse lipid profiles that are not fully captured by standard risk calculators [28,103]. While QRISK4 improves population-level prediction by adding COPD as a risk factor and outperforming earlier versions, it does not explicitly model a history of TB or the synergistic hazards of COPD–TB overlap. Accordingly, in patients with both COPD and prior TB, QRISK4 estimates should be interpreted conservatively, with risk calibration augmented by clinical judgment and consideration of inflammatory and metabolic biomarkers to avoid underestimation of true CVD risk [28,100,102].

Emerging risk stratification approaches extend beyond conventional calculators and appear well suited to patients with COPD–TB overlap. Atherogenic indices derived from routine lipids such as CRI-I, CRI-II, the atherogenic index of plasma (AIP), and the atherogenic coefficient (AC) have classified pulmonary TB cohorts as high cardiovascular risk (CRI-I > 4.21, CRI-II > 3.0) and effectively predicted CVD risk where standard scores may underperform, offering a simple, low-cost signal [103]. CT-based whole-lung radiomics nomograms that fuse quantitative chest CT features with clinical variables such as age, weight, GOLD stage, achieve superior discrimination for CVD risk in COPD (AUC ≈ 0.73) compared with clinical factors alone, and could be adapted to post-TB phenotypes when imaging is available [104]. Finally, machine learning and multimorbidity models such as random forests, ensemble methods leveraging indices like the Pharmacy-based Comorbidity Index, outperform traditional logistic regression for CVD prediction in COPD and can explicitly incorporate TB history to improve discrimination and reclassification [105]. Together, these tools provide complementary lenses metabolic, structural, and integrative that may uncover excess cardiovascular risk in COPD patients with prior TB not fully captured by QRISK4 or other standard scores.

Atherogenic indices, CT-based radiomics nomograms, and machine learning models incorporating multimorbidity offer promising, novel approaches for CVD risk prediction in COPD patients with a history of TB. These tools may better capture the unique risk profile of this population compared to traditional scores.

Regarding the coexistence of COPD and obstructive sleep apnea (OSA), the overlap syndrome is consistently associated with greater nocturnal desaturation, poorer sleep quality, higher cardiovascular comorbidity burden, more frequent exacerbations and hospitalizations, and increased mortality compared with either condition alone, with reported prevalence among COPD cohorts as high as ~66% [106,107]. Overlap patients often present atypically more nocturia, less classic OSA symptoms and exhibit more severe nocturnal hypoxemia and multimorbidity such as CVD, hypertension, diabetes, depression, than single-disease comparators. Early recognition and positive airway pressure therapy reduce exacerbations, admissions, and mortality [106,108].

Although prior pulmonary TB is an established risk factor for COPD and is associated with greater airflow limitation, heightened systemic inflammation, and elevated cardiovascular risk the contemporary literature provides no direct data on the prevalence, phenotype, or outcomes of COPD-OSA overlap specifically among individuals with a TB history [109,110]. Given the more severe structural lung damage and persistent inflammatory milieu typical of TB-related COPD, it is biologically plausible that overlap in this subgroup confers disproportionately higher cardiopulmonary risk, including more profound nocturnal hypoxemia. Mechanistically, the additive burden likely reflects convergent pathways systemic inflammation, oxidative stress, endothelial dysfunction, and autonomic dysregulation, each of which are accentuated in post-TB COPD due to enduring pulmonary and vascular injury. Clinically, overlap syndrome is linked to pulmonary hypertension, more severe COPD exacerbations, increased hospitalizations, and worse survival; by extension, post-TB patients already predisposed to more severe airflow impairment and systemic inflammation may experience amplified morbidity when overlap is present [111,112]. Nevertheless, the absence of targeted epidemiologic and interventional studies in this specific population remains a critical evidence gap and a priority for future research.

## 5. Management Implications for COPD–OSA Overlap in Post-TB Populations

Given the limited prospective validation in post-TB COPD populations, the clinical considerations outlined here should be interpreted as pragmatic, hypothesis-generating proposals rather than guideline-ready recommendations. Most supporting evidence derives from observational studies, extrapolation from COPD or TB populations studied in isolation, and mechanistic plausibility; therefore, implementation should be individualized to patient context and local resources, and future prospective studies are needed to confirm clinical benefit.

While COPD–OSA overlap is an important high-risk phenotype, post-TB COPD also warrants structured assessment and management of cardiovascular and pulmonary vascular complications independent of sleep-disordered breathing, given the persistent inflammatory, endothelial, and prothrombotic sequelae of prior tuberculosis.

Given the limited prospective data specific to post-TB populations, a pragmatic approach may be to combine evidence-based OSA therapies with guideline-directed COPD management; while considering structured cardiometabolic risk assessment and modification, as available evidence suggests increased exacerbation risk and mortality in this subgroup. Positive airway pressure (PAP), particularly CPAP, remains the cornerstone, reducing COPD exacerbations, hospitalizations, and mortality; notably, overlap patients treated with CPAP achieve survival comparable to COPD-only cohorts, whereas untreated overlap is associated with excess mortality and admissions [107,113]. Pending prospective validation in post-TB COPD populations, pharmacotherapy may reasonably be guided by established COPD treatment algorithms typically initiating long-acting bronchodilators (LABA/LAMA) and considering ICS for frequent exacerbations and/or eosinophilic inflammation. Triple therapy (ICS/LABA/LAMA) can be considered in selected high-burden phenotypes, with careful individualized cardiovascular risk–benefit appraisal in light of mixed safety signals [114]. Because overlap confers elevated cardiovascular risk (hypertension, heart failure, arrhythmias), early diagnosis, systematic screening, and integrated management are essential. From a cardiovascular standpoint, COPD–OSA in the post-TB context likely amplifies atherothrombotic and arrhythmic risk, as intermittent hypoxia and sympathetic surges are superimposed on TB-related endothelial dysfunction and a prothrombotic milieu. Accordingly, integrate CVD risk stratification into routine care: baseline ECG and BP, lipid profile and HbA1c, and selective echocardiography when NT-proBNP/sST2 are elevated or right-heart strain is suspected; maintain a low threshold for VTE evaluation when D-dimer is raised or desaturation is disproportionate. In this subgroup, QRISK4 estimates may warrant cautious interpretation, and clinicians may consider integrating longitudinal, multimarker trajectories such as IL-6/hs-CRP/fibrinogen, NT-proBNP/sST2, and D-dimer rather than relying on isolated values when contextualizing preventive strategies; where COPD–OSA overlap is present, optimizing CPAP adherence may help attenuate nocturnal blood pressure surges and vascular stress [115].

Given the prominence of nocturnal hypoxemia, close monitoring and correction including judicious supplemental oxygen are critical to mitigate complications [116].

Beyond overlap-specific interventions, management in COPD patients with a history of TB should include aggressive modification of traditional cardiovascular risk factors such as blood pressure, glycemic control, lipids, smoking cessation, weight management, physical activity, and low-threshold evaluation for pulmonary vascular disease and thrombosis when symptoms are disproportionate to spirometric impairment such as unexplained dyspnea, exertional desaturation, syncope, leg swelling, or pleuritic chest pain. Where feasible, clinicians should also review TB-related treatment history and potential cardiovascular toxicities or interactions including QT-prolonging regimens, drug–drug interactions with antiarrhythmics/anticoagulants and ensure integrated follow-up involving pulmonology and cardiology for patients with recurrent exacerbations, suspected pulmonary hypertension, or prior thromboembolic events. Finally, a personalized, multidisciplinary approach phenotype-driven and attentive to post-TB structural lung damage and systemic inflammation should coordinate pulmonology, sleep medicine, and cardiology to optimize outcomes.

## 6. Diagnostic and Screening Challenges in Resource-Limited Settings

Managing COPD–OSA overlap in LMICs requires pragmatic adaptation to constrained diagnostics, limited device access, and high multimorbidity. Because overlap is frequently underrecognized owing to nonspecific symptoms and scarce polysomnography, programs should deploy validated screening questionnaires (e.g., STOP-Bang, Epworth Sleepiness Scale) to triage high-risk patients for targeted testing, coupled with provider training to improve early recognition and referral [117,118]. CPAP or BiPAP remains the therapeutic cornerstone, reducing exacerbations, hospitalizations, and mortality, but uptake hinges on affordability and availability; LMIC strategies include prioritizing the highest-risk patients, pooled procurement, rental pools, and community-supported adherence.

In resource-limited settings, a pragmatic approach should not only triage sleep-disordered breathing but also systematically screen for cardiovascular disease and TB-related sequelae using low-cost tools. At minimum, this includes standardized blood pressure measurement, diabetes screening (fasting glucose or HbA1c when available), basic lipid testing where feasible, symptom-based assessment for angina/heart failure/stroke, and a resting ECG when accessible. Clinical pathways should incorporate TB history (time since treatment, recurrent disease, and drug exposures) and maintain a low threshold for referral or focused testing when red flags suggest pulmonary hypertension, heart failure, arrhythmia, or venous thromboembolism.

Supplemental oxygen may be used for nocturnal hypoxemia with careful monitoring to avoid CO_2_ retention, while guideline-concordant COPD pharmacotherapy should be optimized with cost-effective long-acting bronchodilators and judicious inhaled corticosteroids [119]. In severe cases or acute decompensation, non-invasive ventilation can be lifesaving where available. At the health-system level, task-shifting to trained non-specialists, risk-based resource allocation, and patient education on symptoms, risk factors, and adherence are essential to extend care into rural and underserved settings and to mitigate the disproportionate cardiopulmonary burden of overlap in LMICs. COPD-OSA patients with a prior history of pulmonary TB constitute a cardiovascular extreme-risk phenotype, in whom intermittent hypoxia and sympathetic surges are superimposed on TB-related endothelial dysfunction and prothrombotic remodeling yielding disproportionate hazards for atherothrombotic events, malignant arrhythmias, heart failure, and venous thromboembolism [63,115].

In LMICs, management of COPD–OSA overlap requires creative adaptation: use of screening tools, prioritization of limited resources, and provider education are key. Expanding access to PAP therapy and optimizing COPD treatment can significantly improve patient outcomes even in resource-constrained settings.

## 7. Unanswered Questions

Key unanswered questions extend beyond COPD-OSA overlap. It remains unclear to what extent prior TB independently amplifies the incidence of major adverse cardiovascular events such as myocardial infarction, stroke, heart failure, and arrhythmias among COPD patients across different regions and exposure strata (e.g., smoking, biomass exposure, HIV, and diabetes). Similarly, the optimal strategy for risk stratification and prevention in post-TB COPD, including whether standard CVD risk scores require recalibration, which biomarkers add meaningful prognostic value, and when to deploy echocardiography or targeted evaluation for venous thromboembolism, requires dedicated prospective studies [13]. Finally, the contribution of TB-related treatment exposures and residual cardiopulmonary pathology to subsequent cardiovascular outcomes in COPD survivors remains insufficiently defined. Second, pathogenic mechanisms linking post-TB structural and immune alterations to COPD, OSA, and their overlap are incompletely resolved; the contributions of chronic inflammation, airway remodeling, and microbiome dysbiosis require focused investigation [15]. Third, there is a shortage of validated, accessible diagnostic pathways and phenotyping strategies suitable for resource-limited settings and for post-TB patients, leaving the optimal approaches for early detection and risk stratification uncertain. Finally, management evidence is sparse: the effectiveness of CPAP, tailored pharmacotherapy, and integrated, multidisciplinary care models on hard outcomes such as exacerbations, hospitalizations, pulmonary hypertension, cardiovascular events, and survival in post-TB overlap populations, particularly in LMICs, remains to be established [15,120].

## 8. Future Directions

The cardiopulmonary burden of COPD–OSA overlap syndrome is well established, but significant gaps remain, especially for post-TB populations. Current research highlights the need for more precise characterization, better diagnostic tools, and targeted interventions. Epidemiology and phenotyping: longitudinal, population-based cohorts are required to define true prevalence, incidence, and risk factors in post-TB groups, coupled with multidimensional phenotyping integrating body shape, comorbidities, symptoms, and wearable data to delineate TB-related overlap endotypes and high-risk subgroups. Pathophysiology and biomarkers: mechanism-anchored studies should interrogate shared and distinct pathways of chronic inflammation, oxidative stress, endothelial dysfunction using molecular, genetic, and biomarker approaches, while parallel discovery and validation programs establish biomarkers for early detection, risk stratification, and longitudinal monitoring. Diagnostics and screening: standardized questionnaires, algorithms, and imaging protocols tailored to post-TB overlap must be developed and validated, with home-based monitoring (blood pressure, nocturnal oximetry, actigraphy) deployed to uncover silent comorbidities and guide adaptive management. Intervention and management: dedicated trials should test positive airway pressure, novel pharmacotherapies, and upper-airway stimulation in post-TB overlap cohorts, alongside integrated care models that address cardiovascular, metabolic, and mental health comorbidities to improve patient-centered outcomes and equity.

## 9. Limitations

This targeted narrative review synthesizes a heterogeneous body of evidence and, by design, did not undertake formal risk-of-bias assessment or meta-analysis. Study-level heterogeneity and residual confounding limit causal inference across several domains. Key effect modifiers smoking intensity (pack-years), biomass exposure (particularly among women), HIV status and antiretroviral therapy, diabetes and glycemic control, nutritional status, socioeconomic conditions/crowding, and altitude were variably measured and inconsistently adjusted for. Few studies provided never-smoker strata or stratified by biomass exposure, and some combined post-TB lung disease with COPD defined by a fixed FEV_1_/FVC threshold rather than lower limit of normal (LLN), raising the risk of diagnostic misclassification. Methodological variability spirometry quality control, DLCO protocols, CT scoring systems and selection processes (clinic-based samples, survivorship and referral biases) may inflate or attenuate effect sizes and complicate cross-region comparisons. Temporality is often uncertain: many data are cross-sectional or retrospective, with limited linkage of biomarkers to hard outcomes. Evidence on COPD–OSA overlap in post-TB populations is largely extrapolated from general COPD cohorts. Finally, our search (2005–2025) and English-language emphasis risk publication and language bias, and the absence of pooled estimates precludes precise quantification of associations.

## 10. Conclusions

TB is a powerful modifier of COPD risk and phenotype. Prior TB independently increases COPD incidence and is associated with more severe disease, including mixed obstructive–restrictive defects, reduced DLCO, and higher exacerbation and hospitalization rates. Persistent inflammation, endothelial dysfunction, thrombosis, and autoimmune/epigenetic remodeling provide biologic plausibility linking post-TB lung disease to atherosclerosis, pulmonary hypertension, and adverse cardiac events; therefore, a vascular-focused assessment may be particularly relevant. Because QRISK4 does not model prior TB or COPD–TB synergy, scores should be interpreted cautiously and complemented, where feasible, by targeted biomarkers and functional metrics alongside standard physiologic testing and focused imaging. In LMICs, pragmatic pathways using simple triage tools, affordable bronchodilators with judicious ICS, prioritized PAP access where indicated, and patient education may improve outcomes.

Future research should prioritize two questions: prospective validation of TB-aware risk stratification beyond conventional risk scores, and pragmatic trials testing whether integrated post-TB COPD care pathways reduce exacerbations and major cardiovascular outcomes versus standard management.

## Figures and Tables

**Table 1:** Summary of observational evidence in post-TB lung disease and COPD outcomes.

Study Region/Country	No of Participants	Prevalence of Prior TB in COPD Cohorts	Prevalence of COPD in Prior TB	Studies
LMICs (multicounty, review)	12,396	2.7% (1.7–15.5%)	25.7%	Katarina Kamenar et al. 2021 [10]
Latin America (multicenter, cross-sectional survey)	5571	7.8–19.7%	30.7%	Menezes A et al. 2005 [25]
India (tertiary-care hospital)	74	32.4%	-	D. Aggarwal et al. 2017 [22]
Northwest China (observational, cross-sectional analysis)	3249	2.7%	12.1%	Yide Wang et al. 2022 [26]
South Africa (population-based BOLD survey)	847	-	49.2%	Allwood B. et. al. 2017 [27]
Egypt (hospital-based)	500	16%	-	Mohamed A. Zamzam et al. 2022 [23]
Romania (multicenter, tertiary-care hospital)	403	64.3%	73.8% (non-smokers) 84.4% (smokers)	Cioboata et al. 2025 [13]
Global (23 studies, meta-analysis)	528,179	-	21% (16–25%)	H. Fan et al. 2021 [28]

## Data Availability

The original contributions presented in this study are included in the article. Further inquiries can be directed to the corresponding authors.
